# Conceptualizing the complexity of ferroptosis to treat triple-negative breast cancer: theory-to-practice

**DOI:** 10.20892/j.issn.2095-3941.2022.0767

**Published:** 2023-03-02

**Authors:** Hang Zhang, Fan Yang, Yi Xiao, Yi-Zhou Jiang, Zhi-Ming Shao

**Affiliations:** Key Laboratory of Breast Cancer in Shanghai, Department of Breast Surgery, Fudan University Shanghai Cancer Center; Department of Oncology, Shanghai Medical College, Fudan University, Shanghai 200032, China

Ferroptosis, a type of regulated cell death named one decade ago, is a unique type driven by lipid peroxidation in an iron-dependent manner. Ferroptosis differs radically from apoptosis and other regulated forms of cell death in both morphology and molecular underpinning. Ferroptosis can be triggered by a variety of physiologic conditions and pathologic stresses. There has been growing interest in ferroptosis in recent years, and research on ferroptosis is productive. The existing evidence has shown that ferroptosis is closely related to cancer initiation, progression, and suppression. Thus, ferroptosis has shown promising potential in cancer therapies. Inducing ferroptosis in tumors and the combination of ferroptosis inducers with other therapies may overcome drug resistance during cancer treatment. Because of the intricate network regulating ferroptosis-related pathways, drugs targeting ferroptosis are diverse and complicated. Thus, deciphering the complexity of ferroptosis and precisely targeting ferroptosis are needed.

## Heterogeneity of ferroptosis-related pathways among tumors

**Figure 1:**
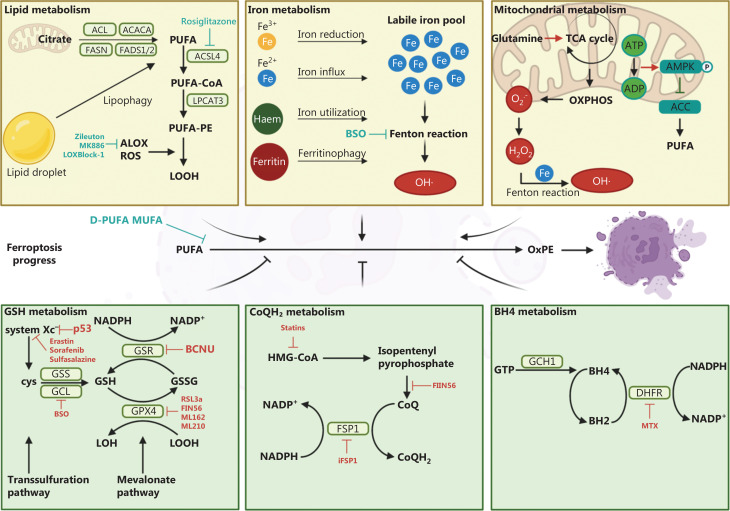
Overview of the regulatory network of ferroptosis. Schematic description of regulated pathways in ferroptosis, including three promoting mechanisms and three defense mechanisms. Inhibitors of ferroptosis-related pathways are included. ATP citrate lyase (ACL); acetyl-CoA (ACACA); fatty acid synthase (FASN); fatty acid desaturase (FADS); polyunsaturated fatty acid (PUFA); acyl-CoA synthetase long-chain family member 4 (ACSL4); lysophosphatidylcholine acyltransferase 3 (LPCAT3); phospholipids containing polyunsaturated fatty acids (PE-PUFAs); lipoxygenase (ALOX); reactive oxygen species (ROS); lipid hydroperoxides (LOOH); hydroxyl radical (OH•); tricarboxylic acid (TCA); oxidative phosphorylation (OXPHOS); hydrogen peroxide (H_2_O_2_); adenosine monophosphate-activated protein kinase (AMPK); acetyl-CoA carboxylase (ACC); oxidized phosphatidylethanolamine (OxPE); glutathione (GSH); glutathione synthase (GSS); glutamic acid-cysteine ligase (GCL); cystine (cys); glutathione disulfide (GSSG); glutathione-disulfide reductase (GSR); lipid alcohol (LOH); ubiquinol (CoQH_2_); ubiquinone (CoQ); 3-hydroxy-3-methylglutarylcoenzyme A (HMG-CoA); ferroptosis suppressor protein 1 (FSP1); GTP cyclohydrolase 1 (GCH1); tetrahydrobiopterin (BH4); dihydrobiopterin (BH2); dihydrofolate reductase (DHFR); deuterated polyunsaturated fatty acid (D-PUFA); monounsaturated fatty acid (MUFA).

Ferroptosis is executed by membrane oxidative damage with increased lipid peroxidation. Peroxidation of polyunsaturated fatty acids (PUFAs) is essential for triggering ferroptosis. Multiple metabolic pathways evolve during the process of ferroptosis. Ferroptosis-promoting pathways include the lipid, iron, and mitochondrial metabolism pathways (**Figure 1**). PUFA peroxidation can occur through a series of enzyme catalysis in the lipid metabolism pathway. Acyl-CoA (coenzyme) synthetase long-chain family member 4 (ACSL4) catalyzes the ligation of PUFAs with CoA and generates CoA-PUFAs. CoA-PUFAs are esterified by lysophosphatidylcholine acyltransferase 3 (LPCAT3) to form phospholipids containing polyunsaturated fatty acids (PE-PUFAs). PE-PUFAs are mainly catalyzed by autoxidation and are prone to peroxidization by lipoxygenase (ALOX) or cytochrome P450 oxidoreductase (POR) into lipid hydroperoxides. The generation of lipid hydroperoxides promotes ferroptosis initiation. Iron metabolism has multiple processes, including iron absorption, storage, utilization, and efflux. Indeed, cells maintain a relatively labile iron pool *via* these processes; however, cancer cells have dysregulated iron metabolism and an increased labile iron pool. Iron increases ALOX activity. The free ferrous iron in the labile iron pool also participates in the Fenton reaction to generate free radicals and mediate lipid peroxidation. Mitochondrial metabolism is another pathway that promotes ferroptosis. Mitochondria, as the site of multiple metabolic pathways in the cell, are the main source of cellular reactive oxygen species (ROS). The electrons leaked from the electron transport chain (ETC) complexes can be used to generate hydrogen peroxide (H_2_O_2_). H_2_O_2_ reacts with ferrous iron to generate hydroxyl radicals (OH•), which leads to lipid peroxidation^1^. In addition, anaplerotic reactions that replenish the tricarboxylic acid (TCA) cycle, such as glutaminolysis, promote ferroptosis by increasing fatty acid biosynthesis and electron leakage^1^.

Cells have developed ways to defend against ferroptosis, including the glutathione (GSH), ubiquinol (CoQH_2_), and tetrahydrobiopterin (BH4) metabolism pathways (**Figure 1**). Cancer cells obtain cystine through System Xc^-^, which is embedded in the cell membrane, during GSH metabolism. Then, cystine is reduced to cysteine and catalyzed by glutamic acid-cysteine ligase (GCL) and glutathione synthase (GSS) to synthesize GSH. Glutathione peroxidase 4 (GPX4), a regulator of ferroptosis, uses GSH as a cofactor to catalyze lipid peroxide reduction and prevent ferroptosis. The FSP1 (ferroptosis suppressor protein 1)-CoQH_2_ axis is another pathway that prevents ferroptosis. FSP1 is recruited to the plasma membrane, acts as an oxidoreductase, and reduces ubiquinone (known as CoQ) to CoQH_2_, which traps lipid peroxyl radicals and inhibits lipid peroxidation. The BH4-DHFR (dihydrofolate reductase) axis is a GPX4-independent mechanism for ferroptosis regulation. BH4 is an antioxidant capable of trapping lipid peroxide-free radicals. BH4 undergoes redox cycling through DHFR to protect lipid membranes from autoxidation. BH4 synthesis is a critical pathway involved in GPX4 inhibition.

Energy-related metabolism also has a role in regulating ferroptosis. Several cellular energy metabolism pathways including glycolysis, pentose phosphate pathway (PPP) and TCA cycle are closely associated with oxidized phosphatidylethanolamine (OxPE) biosynthesis and the generation of reducing substances. Mitochondria, as the energy powerhouse in cells, coordinate various metabolic processes and have a key role in ferroptosis. Mitochondria generate ROS to execute ferroptosis *via* canonical metabolic processes, including the TCA cycle and mitochondrial ETC^1^. Tumor cells have a higher glycolytic rate and greater suppression of oxidative phosphorylation (OXPHOS) activity than non-tumor cells^2^. Therefore, alleviating ROS stress can prevent ferroptosis in tumor cells. Due to excessive consumption of adenosine triphosphate (ATP) in tumor cells, the cellular energy sensor adenosine monophosphate-activated protein kinase (AMPK) is activated^2^. AMPK inhibits the ability of the acetyl-CoA carboxylases, ACC1/ACC2, to maintain the nicotinamide adenine dinucleotide phosphate hydrogen (NADPH) level. ACC inhibition results in suppression of PUFA synthesis, thus leading to ferroptosis resistance. The PPP is a method by which glucose is oxidatively decomposed. Cancer cells exhibit enhanced PPP activity. PPP utilizes glucose 6-phosphate (G6P) and generates ribose 5-phosphate, erythrose 4-phosphate, and NADPH. NADPH donates electrons for the reduction of glutathione disulfide (GSSG) to GSH, which supports the regeneration of thioredoxin (Trx) and cooperates with FSP1 to reduce CoQ to CoQH_2_, thus preventing cells from undergoing ferroptosis. In addition, ferroptosis is genetically regulated. As the most common mutated genes in tumors, p53 and RAS (KRAS, NRAS and HRAS) are associated with ferroptosis. p53 inhibits cystine uptake *via* transcriptional suppression of the cystine/glutamate antiporter solute carrier family member 11 (SLC7A11) and sensitizes cells to ferroptosis^3^. On the other hand, p53 inhibits erastin-triggered ferroptosis by suppressing dipeptidyl peptidase-4 (DPP-4) activity^4^. As ferroptosis was originally found in cells expressing the mutant RAS oncogene, there is also a correlation between the RAS oncogene and ferroptosis. However, the intrinsic mechanism is complicated and still needs further exploration.

Because of the complex regulatory network in ferroptosis and the high heterogeneity of tumors, sensitivity to ferroptosis varies greatly between different subtypes of tumors. Thus, depicting the ferroptosis landscape in tumors can help us better understand diseases and develop novel targeted therapy strategies.

## Remodeling of the tumor microenvironment after triggering ferroptosis

Ferroptosis is immunogenic *in vitro* and *in vivo*^5^. The immune system has a substantial role in exerting antitumor immunity; however, tumor cells have developed multiple ways to escape immune surveillance, including reducing immunogenicity and forming immunosuppressive networks, rendering tumor immunotherapy clinically inefficient^6^. Therefore, inducing ferroptosis in tumor cells stimulates the immune system and enhances the efficacy of immunotherapy. Immunogenic cell death (ICD) is a type of regulated cell death (RCD) in which damage-associated molecular patterns (DAMPs) are released to promote antitumor immunity. ATP and high mobility group box 1 (HMGB1), as DAMPs, are released by early ferroptotic cancer cells and serve as immunogenic signals to stimulate antigen-presenting cells (APCs)^5^. Ferroptotic cells, like ICD, release ‘find me’ signals to recruit APCs and other immune cells to the ferroptotic microenvironment. Arachidonic acid oxidation products released by ferroptotic cells activate antitumor immunity; however, oxidized lipids are also associated with inhibition of antitumor immune responses. The accumulation of oxygenated neutral lipids and PUFAs in dendritic cells (DCs) results in defective cross-presentation and poor CD8^+^ T-cell stimulation^7^. In addition to lipid signaling, ferroptotic cancer cells release HMGB1 in an autophagy-dependent manner. HMGB1 belongs to the DAMP family and binds to Toll-like receptor 4 (TLR4) and advanced glycosylation end product-specific receptor (AGER) to modulate the immune response. HMGB1 accelerates the phagocytic cargo ability in DCs and promotes antigen presentation to T cells^8^. Although ferroptotic tumor cells stimulate antitumor immune capability, some substances released by ferroptotic tumor cells also suppress immunity. Release of 8-hydroxyguanosine (8-OHG) by ferroptotic cells leads to macrophage infiltration and promotes pancreatic ductal adenocarcinoma (PDAC) tumorigenesis in mice^9^. Prostaglandin E2 (PGE2) released by ferroptotic cells suppresses the antitumor function of immune cells and causes tumor immune escape, leading to disease progression^10^.

In addition to tumor cells, the tumor microenvironment is a very large system with various kinds of immune cells that are affected by ferroptosis. CD8^+^ T cells, as critical antitumor immune cells, exhibit high susceptibility to GPX4 inhibitor-induced ferroptosis due to the massive lipid peroxides^11^. Studies have shown that CD8^+^ T cells exhibit greater sensitivity to GPX4 inhibitors than tumor cells and ACSL4 gene deletion protects CD8^+^ T cells from ferroptosis^11^. Regulatory T cells (Tregs) have a role in suppressing antitumor immunity. Unlike CD8^+^ T cells, Tregs carry low amounts of lipid peroxide and exhibit hyposensitivity to ferroptosis^11^. This finding may be because GPX4 in Tregs prevents them from undergoing lipid peroxidation and ferroptosis. Specific deletion of GPX4 in Tregs results in lipid peroxide accumulation and ferroptosis of Tregs, suppresses tumor growth, and enhances antitumor immunity^12^. Tumor-associated macrophages (TAMs) are classified as M1 (pro-inflammatory) and M2 (anti-inflammatory) phenotypes. Although the expression of several known anti-ferroptotic pathways is comparable between M1 and M2 macrophages, M1 macrophages exhibit resistance to ferroptosis inducers. Higher levels of inducible NO synthase (iNOS or NOS2) and NO• are detected in M1 macrophages than M2 macrophages^13^. Myeloid-derived suppressor cells (MDSCs) are a group of immature myeloid cells with strong anti-T-cell activity that suppress antitumor immunity and promote tumor progression. MDSCs overexpress neutral ceramidase N-acylsphingosine amidohydrolase (Asah2), which catalyzes sphingolipid metabolism to resist ferroptosis^14^. Minimal arachidonic acid-phosphatidylethanolamine (AA-PEox) is measurable in tumor monocytic MDSCs (M-MDSCs), indicating low ferroptosis activity. In contrast, pathologically-activated neutrophils (PMNs), termed myeloid-derived suppressor cells (PMN-MDSCs), have high sensitivity to ferroptosis with downregulation of GPX4 and AA-PEox accumulation. The release of oxygenated lipids and PGE2 by ferroptotic PMN-MDSCs suppresses the activity of T cells^10^. Natural killer (NK) cells are essential for antitumor immunity. A recent study showed that tumor-associated NK cells are enriched in lipid peroxidation-, oxidative damage- and ferroptosis-related pathways, which impair tumor-associated NK cell cytotoxicity^15^. DCs initiate T-cell-mediated immune responses. The increased level of lipids disrupts the antigen processing function of tumor-associated DCs^7^. Thus, ferroptosis is heterogeneous in different tumor microenvironment cells. Inducing ferroptosis inhibits the activity of antitumor immune cells by increasing cell death. Moreover, ferroptosis kills immunosuppressive cells. Therefore, ferroptosis induction of immunosuppressive cells achieves an antitumor effect.

Because ferroptosis is immunogenic, inducing ferroptosis is a potential approach for promoting cancer immunotherapy. Various studies have explored targeting ferroptosis in combination with immunotherapy. BEBT-908 is a dual-targeting phosphoinositide 3-kinase (PI3K) and histone deacetylase (HDAC) inhibitor that induces immunogenic ferroptosis in cancer cells. Combining BEBT-908 with anti-PD1 therapy potently inhibits tumor cell growth and proliferation^16^. The tyrosine-protein kinase receptor (TYRO3) inhibitor, LDC1267, and the GPX4 inhibitor, RSL3, improve the efficacy of immune checkpoint inhibitor (ICI) therapy^17^. Beyond traditional ferroptosis inducers, nanoplatforms have been applied to design ferroptosis nanoparticle inducers for specific drug delivery. The combination of nanoparticles encapsulating RSL-3 with anti-PD 1 therapy significantly inhibits the growth of 4T1 breast cancer and melanoma cells. Thus, the combination strategies of ferroptosis inducers with immunotherapy are diverse, and tumors with different ferroptosis-related features should be treated with different ferroptosis-related therapies. In our most recent study, we demonstrated that luminal androgen receptor (LAR) tumors are particularly sensitive to the GPX4 inhibitor, RSL-3. An inflammatory phenotype occurs after ferroptosis induction, which provides the possibility of combining ferroptosis with immunotherapy^18^.

## Precisely targeting ferroptosis for therapy through a holistic tailored approach

As previously illustrated, ferroptosis has diverse regulatory pathways, complicated networks, and multiple targeting options. As a result, different tumors might utilize different mechanisms to regulate ferroptosis activity. Therefore, it is important to analyze ferroptosis from a holistic approach in the beginning and select individualized drugs to target ferroptosis; however, current research on ferroptosis lacks a holistic view. Several studies involving lung cancer^19^, hepatocellular carcinoma (HCC)^20^, and pancreatic cancer^21^ have only focused on a single gene and detailed mechanisms, and did not consider that ferroptosis inducers act on tumor cells and have an impact on the immune microenvironment. Therefore, the value of ferroptosis inducers for clinical application is limited.

From a clinical perspective, it is important to systematically analyze ferroptosis-related features in tumors. Identifying suitable drug candidates can provide new strategies for cancer treatment. Triple-negative breast cancer (TNBC) is a biologically and clinically heterogeneous disease. It is important to identify which subtype of TNBC is sensitive to ferroptosis and which drug targeting ferroptosis is most suitable.

In our recent study we integrated pathway analysis of transcriptomic data and key metabolite analysis of metabolomic data, and utilized TNBC cell lines and samples for validation to reveal ferroptosis heterogeneity in TNBC. Our multiomic analysis has never been performed. We discovered that TNBCs have heterogeneous phenotypes in ferroptosis-related pathways and metabolites. We focused on LAR TNBCs, which are enriched in ferroptosis-related pathways, and concluded that GSH metabolism is critical for suppressing ferroptosis in LAR tumors. We selected TS/A, a mouse LAR tumor cell line, to establish an orthotopic model. Using the GPX4 inhibitor, RSL3, we observed pronounced ferroptosis in LAR tumor cells. In clinical translation, using a GPX4 inhibitor enhanced anti-PD 1 therapy efficacy in mice, and the combination of RSL3 and anti-PD 1 therapy reached a synthesized effect. We performed immunohistochemistry (IHC) staining and flow cytometric analysis after *in vivo* experiments. Specifically, we observed direct changes in the tumor microenvironment after using a ferroptosis inducer in this tumor subtype with the recruitment of CD3e^+^, CD4^+^, CD8^+^, and CD86^+^ cells, and a reduced number of CD206^+^ cells. When combined with immune checkpoint blockade (ICB) therapy, the cytotoxicity of CD8+ T cells is improved. Thus, a ferroptosis inducer can be optimal therapy using GPX4 inhibitors with ICB in LAR tumors^18^. In conclusion, our previous study systematically analyzed the ferroptosis characteristics of TNBC from a holistic view and validated the ferroptosis-sensitive subtype in TNBC. The combination therapy we proposed has high clinical translation value. Furthermore, our approach can provide new ideas for other ferroptosis-related studies. In terms of method innovation, previous research has mainly focused on a single gene and detailed mechanisms, lacking a holistic view of the ferroptosis features in tumors. In our study we performed a multiomic analysis to reveal and validate heterogeneous TNBC ferroptosis phenotypes in ferroptosis-related pathways and metabolites. With respect to clinical applications, some studies have summarized and reviewed ferroptosis-related drugs that can improve immunotherapy efficacy; however, tumors with different ferroptosis-related features are suitable for different ferroptosis-related therapeutic strategies. We first used GPX4 inhibitors to induce tumor immunogenicity and showed that LAR tumors are hypersensitive to GPX4 inhibitors. Moreover, at the conceptual level, although previous studies have indicated a close link between androgen receptor (AR) and GPX4, none of the studies elaborated a clear regulatory mechanism of AR on GPX4. In our study we further investigated the mechanism underlying AR on GPX4 and used the mechanism to further explain the ferroptosis characteristics of LAR tumors. Nonetheless, our study had some limitations. The treatment strategy we proposed is only applicable to LAR subtype patients, which represents a small number of breast cancer patients^22^. In future corollary studies, we will investigate the ferroptosis characteristics of other breast cancer molecular types and provide possible treatment strategies to benefit more patients. In addition, our study did not identify biomarkers of ferroptosis treatment, which also warrants investigation.

## Conclusions

Collectively, ferroptosis, as one of the RCD types with various regulated pathways, provides us with a novel approach in cancer treatment. The immunogenic features of ferroptosis provide the potential for combining ferroptosis inducers with immunotherapy treatments; however, due to the heterogenicity and impact on the immune microenvironment, it is important to study ferroptosis from a holistic view in future research. Our previous study demonstrated ferroptosis heterogeneity in TNBC, validated a ferroptosis-sensitive TNBC subtype, and proposed a strategy combining ferroptosis inducers with anti-PD-1 therapy that showed innovative potential in clinical application, thus providing a model for other ferroptosis-related studies.

## References

[1] Gan B (2021). Mitochondrial regulation of ferroptosis. J Cell Biol.

[2] Yao X, Li W, Fang D, Xiao C, Wu X, Li M (2021). Emerging roles of energy metabolism in ferroptosis regulation of tumor cells. Adv Sci (Weinh).

[3] Jiang L, Kon N, Li T, Wang SJ, Su T, Hibshoosh H (2015). Ferroptosis as a p53-mediated activity during tumour suppression. Nature.

[4] Xie Y, Zhu S, Song X, Sun X, Fan Y, Liu J (2017). The tumor suppressor p53 limits ferroptosis by blocking DPP4 activity. Cell Rep.

[5] Efimova I, Catanzaro E, van der Meeren L, Turubanova VD, Hammad H, Mishchenko TA (2020). Vaccination with early ferroptotic cancer cells induces efficient antitumor immunity. J Immunother Cancer.

[6] Wang W, Zhang L, Sun Z (2022). Eliciting pyroptosis to fuel cancer immunotherapy: mechanisms and strategies. Cancer Biol Med.

[7] Veglia F, Tyurin VA, Mohammadyani D, Blasi M, Duperret EK, Donthireddy L (2017). Lipid bodies containing oxidatively truncated lipids block antigen cross-presentation by dendritic cells in cancer. Nat Commun.

[8] Yamazaki T, Hannani D, Poirier-Colame V, Ladoire S, Locher C, Sistigu A (2014). Defective immunogenic cell death of HMGB1-deficient tumors: compensatory therapy with TLR4 agonists. Cell Death Differ.

[9] Dai E, Han L, Liu J, Xie Y, Zeh HJ, Kang R (2020). Ferroptotic damage promotes pancreatic tumorigenesis through a TMEM173/STING-dependent DNA sensor pathway. Nat Commun.

[10] Kim R, Hashimoto A, Markosyan N, Tyurin VA, Tyurina YY, Kar G (2022). Ferroptosis of tumour neutrophils causes immune suppression in cancer. Nature.

[11] Drijvers JM, Gillis JE, Muijlwijk T, Nguyen TH, Gaudiano EF, Harris IS (2021). Pharmacologic screening identifies metabolic vulnerabilities of CD8(+) T cells. Cancer Immunol Res.

[12] Xu C, Sun S, Johnson T, Qi R, Zhang S, Zhang J (2021). The glutathione peroxidase Gpx4 prevents lipid peroxidation and ferroptosis to sustain Treg cell activation and suppression of antitumor immunity. Cell Rep.

[13] Kapralov AA, Yang Q, Dar HH, Tyurina YY, Anthonymuthu TS, Kim R (2020). Redox lipid reprogramming commands susceptibility of macrophages and microglia to ferroptotic death. Nat Chem Biol.

[14] Zhu H, Klement JD, Lu C, Redd PS, Yang D, Smith AD (2021). Asah2 represses the p53-Hmox1 axis to protect myeloid-derived suppressor cells from ferroptosis. J Immunol.

[15] Poznanski SM, Singh K, Ritchie TM, Aguiar JA, Fan IY, Portillo AL (2021). Metabolic flexibility determines human NK cell functional fate in the tumor microenvironment. Cell Metab.

[16] Fan F, Liu P, Bao R, Chen J, Zhou M, Mo Z (2021). A dual PI3K/HDAC inhibitor induces immunogenic ferroptosis to potentiate cancer immune checkpoint therapy. Cancer Res.

[17] Zhao YY, Lian JX, Lan Z, Zou KL, Wang WM, Yu GT (2021). Ferroptosis promotes anti-tumor immune response by inducing immunogenic exposure in HNSCC. Oral Dis.

[18] Yang F, Xiao Y, Ding JH, Jin X, Ma D, Li DQ (2022). Ferroptosis heterogeneity in triple-negative breast cancer reveals an innovative immunotherapy combination strategy. Cell Metab.

[19] Zhang W, Sun Y, Bai L, Zhi L, Yang Y, Zhao Q (2021). RBMS1 regulates lung cancer ferroptosis through translational control of SLC7A11. J Clin Invest.

[20] Yao F, Deng Y, Zhao Y, Mei Y, Zhang Y, Liu X (2021). A targetable LIFR-NF-κB-LCN2 axis controls liver tumorigenesis and vulnerability to ferroptosis. Nat Commun.

[21] Kremer DM, Nelson BS, Lin L, Yarosz EL, Halbrook CJ, Kerk SA (2021). GOT1 inhibition promotes pancreatic cancer cell death by ferroptosis. Nat Commun.

[22] Ding R, Xiao Y, Mo M, Zheng Y, Jiang YZ, Shao ZM (2022). Breast cancer screening and early diagnosis in Chinese women. Cancer Biol Med.

